# Use of baseline 18F-FDG PET scan to identify initial sub-volumes with local failure after concomitant radio-chemotherapy in head and neck cancer

**DOI:** 10.18632/oncotarget.25030

**Published:** 2018-04-24

**Authors:** Floriane Legot, Florent Tixier, Minea Hadzic, Thomas Pinto-Leite, Christelle Gallais, Rémy Perdrisot, Xavier Dufour, Catherine Cheze-Le-Rest

**Affiliations:** ^1^ Department of Nuclear Medicine, Poitiers University Hospital, Poitiers, France; ^2^ Radiotherapy Department, Poitiers University Hospital, Poitiers, France; ^3^ Head and Neck Department, Poitiers University Hospital, Poitiers, France

**Keywords:** 18F-FDG PET, head and neck cancer, recurrence

## Abstract

**Introduction:**

Head and neck squamous cell carcinoma (HNSCC) treated by radio-chemotherapy have a significant local recurrence rate. It has been previously suggested that 18F-FDG PET could identify the high uptake areas that can be potential targets for dose boosting. The purpose of this study was to compare the location of initial hypermetabolic regions on baseline scans with the metabolic relapse sites after radio-chemotherapy in HNSCC.

**Results:**

The initial functional tumor volume was significantly higher for patients with proven local recurrence or residual disease (23.5 cc vs. 8.9 cc; *p* = 0.0005). The overlap between baseline and follow-up sub-volumes were moderate with an overlap fraction ranging from 0.52 to 0.39 between R40 and I30 to I60.

**Conclusion:**

In our study the overlap between baseline and post-therapeutic metabolic tumor sub-volumes was only moderate. These results need to be investigated in a larger cohort acquired with a more standardized patient repositioning protocol for sequential PET imaging.

**Methods:**

Pre and post treatment PET/CT scans of ninety four HNSCC patients treated with radio-chemotherapy were retrospectively reviewed. Follow-up 18F-FDG PET/CT images were registered to baseline scans using a rigid body transformation. Seven metabolic tumor sub-volumes were obtained on the baseline scans using a fixed percentage of SUV_max_ (I30, I40, I50, I60, I70, I80, and I90) and were subsequently compared with two post-treatment sub-volumes (R40, R90) in 38 cases of local recurrence or residual metabolic disease. Overlap fraction, Dice and Jaccard indices, common volume/baseline volume and common volume/recurrent volume were used to determine the overlap of the different estimated sub-volumes.

## INTRODUCTION

Head and neck squamous cell carcinoma (HNSCC) is the eighth most common cancer worldwide with over half a million new cases diagnosed annually [1]. Approximately two thirds of HNSCCs are already advanced at diagnosis with a 5 year survival below 50%. High mortality rate is largely due to recurrence occurring in the majority of the cases locally and within the first two years after curative treatment. The current treatment of choice of inoperable advanced HNSCCs is concomitant radio-chemotherapy. Advanced HNSCC are usually large, heterogeneous with hypoxic components, thus requiring theoretical doses up to 90 Gy [2, 3, 4], not conceivable in practice given the surrounding normal organs at risk. New radiotherapy techniques such the intensity-modulated radiotherapy (IMRT) can be used nowadays to better target the tumor [5]. Reducing target volumes is expected to allow dose escalation while preserving healthy surrounding tissues and thus potentially increase local control without impairing tolerance [6].

Currently 18F-FDG PET is performed at the time of diagnosis to identify involved lymph nodes or synchronous tumors, but these baseline images can be equally used for radiotherapy planning purposes [7]. In 2004, Daisne *et al.* have shown that the use of 18F-FDG PET images for tumor delineation already led to macroscopic target volumes smaller than the ones derived from CT or MRI data, with a very strong anatomopathological correlation [8]. Few studies have reported that the majority of head and neck cancer failures map to the pretreatment PET abnormalities [9]. These observations suggest that baseline 18F-FDG PET/CT might be useful in detecting intratumor regions of increased radiation resistance. Recent studies hypothesize that failures may arise from the most FDG avid intra-tumor regions. These studies have indeed shown that the sub-volumes with high 18F-FDG uptake tend to correlate with a higher risk of local recurrence after a treatment by radiochemotherapy in non-small cell lung cancer [10, 11], in rectal cancer [12] and esophageal cancer [13]. In head and neck cancer, the only study available reported a less strong correlation when considering a limited group of 19 patients with a local relapse [14]. In addition, this study considered a very heterogeneous group of patients in terms of stage (II-IV), treatment (chemotherapy followed by combined chemo-radiotherapy, radiotherapy, combined chemo-radiotherapy) and disease localization (63% oropharynx) [14].

In this context, our objective was to analyze in a larger and uniform patient cohort the location of residual or recurrent metabolic disease compared to the more active baseline intra-tumor regions of locally advanced head and neck cancer treated by radiochemotherapy, given that those active tumor sub-volumes identified on the 18F-FDG PET images could be preferentially considered for dose escalation.

## RESULTS

### Patient characteristics

The mean follow-up was 38.3 ± 21 months. At the time of analysis, 49 patients were still alive, 2 had missing follow up information and 43 had died from the disease. Of the 94 patients studied, 35 remained in complete response. Out of 29 patients without an initial complete response, 25 had a local residual disease. During the follow-up, 17 patients had a distant relapse with metastatic or nodal disease but without local relapse. Thirteen patients had a suspicion of local recurrence (LR) which was confirmed either histologically (10 patients) or by clinical and/or imaging follow up (3 patients). We considered metabolically active residual disease and local relapse as equivalent for the purpose of this study.

There were no significant differences in age, sex or TNM stage in patient groups according to the presence or not of local relapse. Initial FDG uptake intensity assessed by SUV_max_ was not significantly different in patients with or without local relapse (16.1 ± 5.2 vs. 13.2 ± 3.1, *p* > 0.05). In contrast, MTV measured on baseline scans were significantly higher (*p* = 0.0005) for patients with a local recurrence (median I_40_: 23.5 cm^3^, range: 8.3–65.3) than for patients achieving a complete response (median I_40_: 8.9 cm^3^, range: 3.1–26.1). The mean recurrence MTV using a 40% threshold (R_40_) was 25.4 ± 17.3 cm^3^. Initial and recurrence MTVs measured using different thresholds for patients with residual disease or recurrence (*n* = 38) are detailed in Table 1. Finally, TLG was higher in patients with relapse or residual disease than in patients with a complete response (162 ± 144.7 vs. 98.5 ± 86.5, *p* = 0.01).

**Table 1:** Mean metabolic tumor sub-volumes (cc) obtained using various fixed percentage threshold of SUV_max_ on baseline PET/CT and on follow-up PET/CT for patients with local recurrence or residual disease (*n* = 38)

	30%	40%	50%	60%	70%	80%	90%
Baseline PET/CT	34.1 ± 20.3	24.6 ± 15.1	16.9 ± 11.4	10.8 ± 7.5	6.1 ± 4.9	2.5 ± 2.3	0.61 ± 0.59
Follow-up PET/CT		25.4 ± 17.3					0.18 ± 0.12

### Overlaps between initial and recurrence MTVs

**Figure 1:**
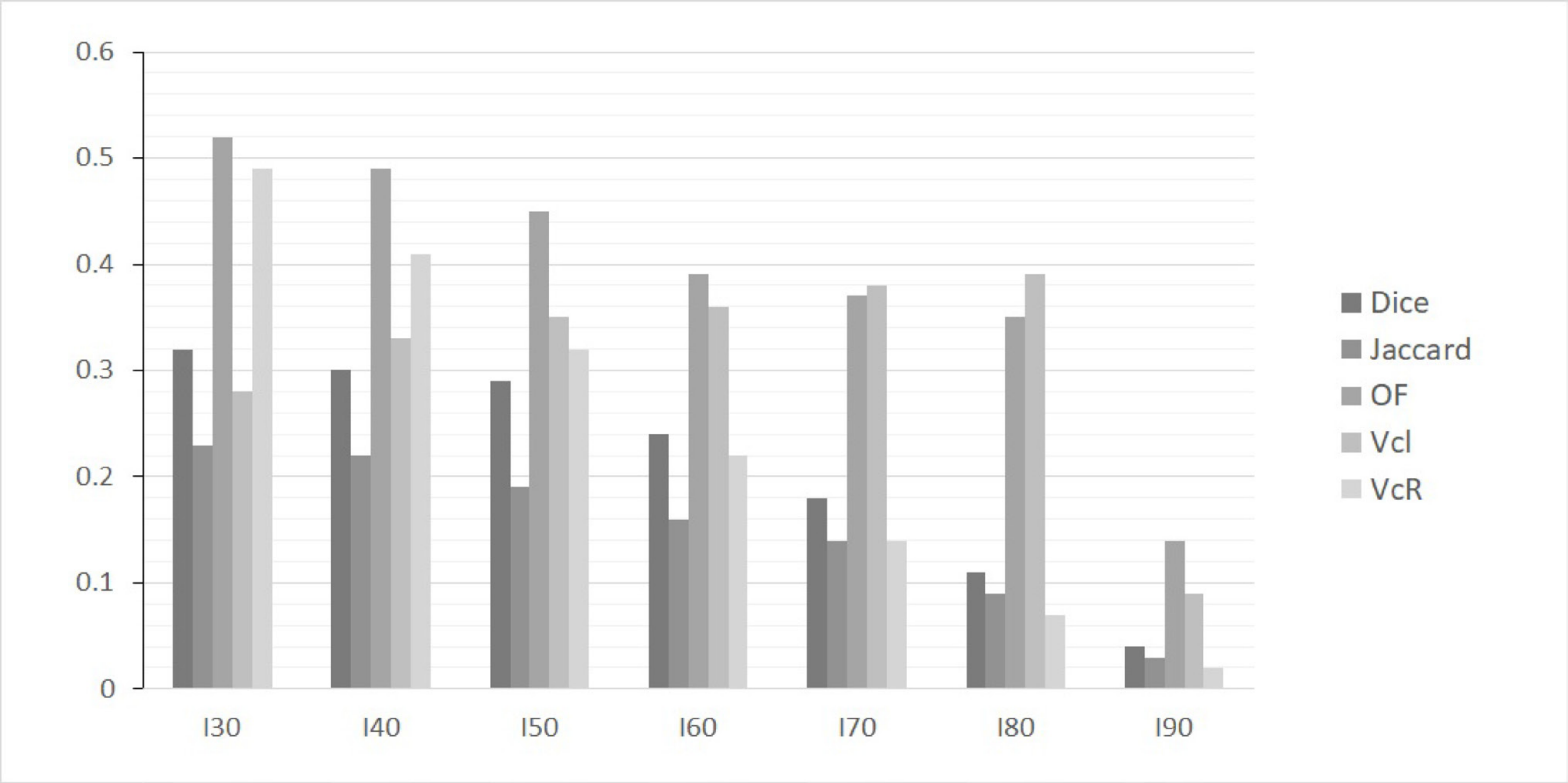
Histogram of mean values of overlap indices for various SUV_max_ thresholds used to delineate sub-volumes on baseline PET/CT (I30, I40, I50, I60, I70, I80 and I90) with the sub-volume R40 measured on follow-up PET/CT images in case of local recurrence or residual disease

**Figure 2:**
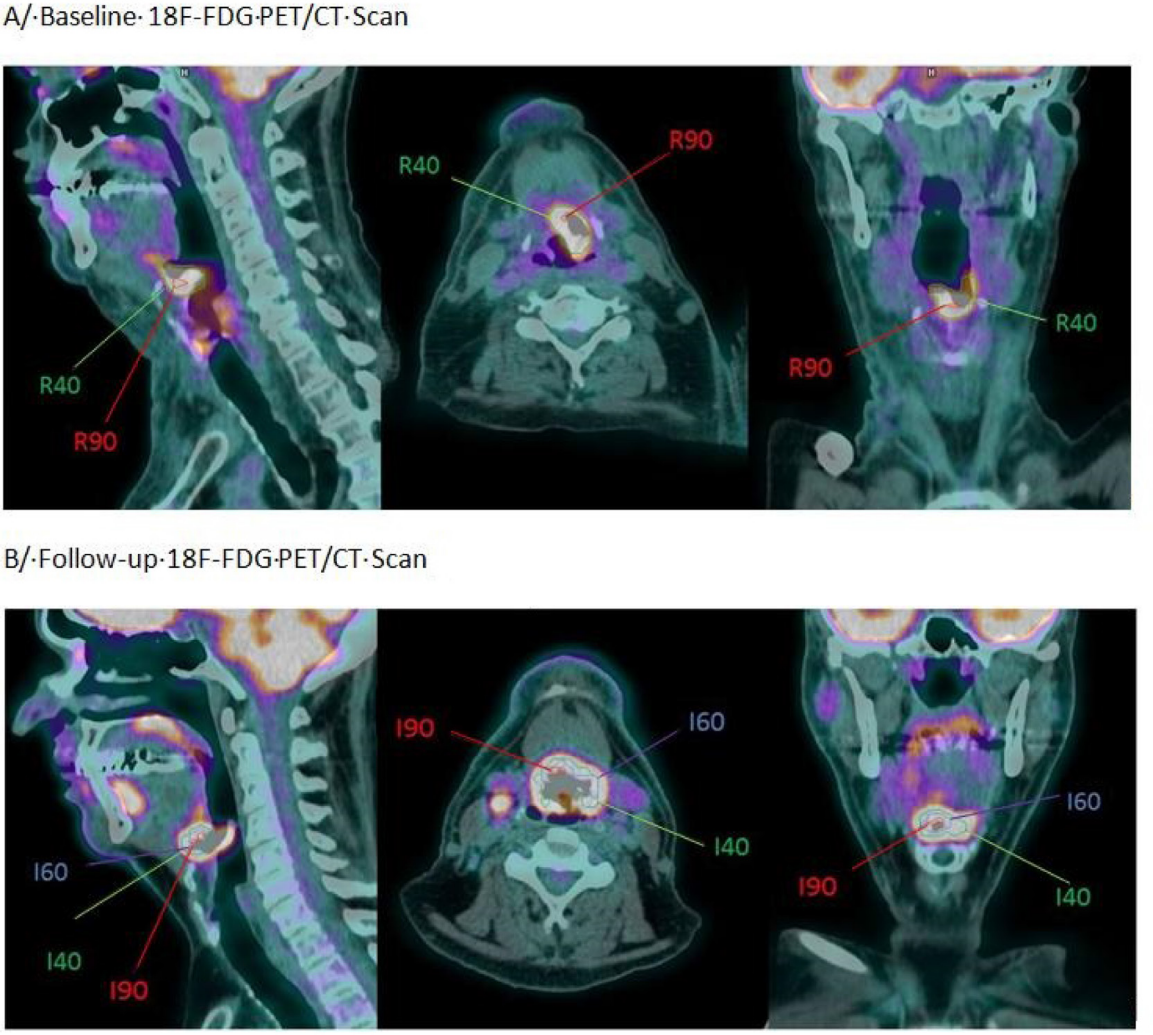
Example of an oropharyngeal cancer (stage T4N2M0) in a 54-year-old patient with residual disease (**A**) Baseline PET/CT Scan (pre-treatment). (**B**) Post-treatment PET/CT scan demonstrating a local recurrence.

Overlap comparisons were performed for all LR patients. A moderate overlap was found between the recurrence MTV provided by a 40% threshold (R_40_) and the initial MTV I_x_. For all I_x_ measurements this value was under 0.55 (Figure 1). In addition, considering the OF between R_40_ and I_x_, we identified a decreasing overlap for increasing *x* value. However, this OF remained >0.35 for overlaps between I_30_, I_40_, I_50_ and I_60_ (Figure 1). Similarly, V_cI_ showed a fair overlap only with values remaining between 0.3 and 0.4 for the comparison of R_40_ with I_40_, I_50_, I_60_, I_70_ and I_80_. A typical patient example is shown in Figure 2.

Considering the intense areas of the baseline images (I_50_ to I_80_), the overlap was similar (V_cI_ < 0.4), dropping to < 0.15 for the most intense regions (I_90_). These highest FDG uptake areas on the baseline PET images (I_90_) were very small with a mean MTV of 0.61 ± 0.59 cm^3^. There was no significant overlap with the recurrence volume R_40_. Considering V_cR,_, the overlap concordance was increasingly lower for initial higher intensity areas at the exception of the comparisons between R_40_ and I_30_ or I_40_ (>0.4). The Jaccard and Dice indices were consistently lower than 0.35 irrespective of the considered thresholds.

Considering the overlap between *R*_*90*_ *and I*_*x*_, the mean volume of high uptake areas in cases of local relapse (R_90_) was also very small (0.18 ± 0.12 cm^3^). In addition, for the majority of the patients there was no overlap between R_90_ and I_x_. A limited overlap was only found in 25% of the patients (5/20) when considering an initial volume defined using a threshold between 30% and 60% (Table 2).

**Table 2:** Number of patient with an overlap between various sub-volumes on baseline PET(I_x_) and R90 in case of residual disease or local recurrence (*n* = 38)

	I30	I40	I50	I60	I70	I80	I90
Number of patients	14 (37%)	13 (34%)	8 (21%)	4 (11%)	2 (5%)	0 (0%)	0 (0%)

## DISCUSSION

Previous studies have reported local or regional recurrence rates between 15% and 35% for HN cancer patients after treatment with curative intent including surgery, chemotherapy or radiotherapy [15]. Regarding the subgroup of patients with locally advanced disease treated by radiotherapy, Oksuz *et al.* have reported a recurrence rate of 12% based on systematic follow up using CT scans [16]. With a mean follow-up of 38 months, we have recorded 17% distant relapses with metastatic or nodal disease but without a local relapse. In addition, for 38% of our patients, the local response was either partial or they experienced a local recurrence. Since our primary objective in this retrospective study was to compare pre and post therapeutic FDG PET /CT scans, our patient selection process naturally focused on patients who benefited from a post therapeutic PET/CT examination. Since post therapeutic PET/CT scans are generally only prescribed, according to current recommendations in case of a clinical suspicion of recurrence [17], the a priori probability to have a positive PET scan after treatment was probably higher in our cohort than in a more general patient population. This selection bias may explain the higher rate of residual or recurrent disease in our cohort. It is now established that among HNSCC, two entities should be considered according to their differential outcome. Tumors non related to human papillomavirus (HPV) are associated with a worse prognosis and a more frequent incomplete response to combined radiochemotherapy [18]. In our study, we did not consider the HPV status, since our main purpose was to study the location and not the cause of the residual disease.

The main objective of our study was indeed to identify on baseline PET/CT images the intra-tumor areas with the higher recurrence risk as demonstrated by a post therapy acquired image. The identification of these areas could translate into potential target volumes to consider for the adaptation of the irradiation planning especially with new radiotherapy techniques such as IRMT [19]. Within this context this study was performed on a group of 94 patients with locally advanced head and neck squamous cell carcinomas, who received a curative intent treatment including radiotherapy ± chemotherapy, and 38 paired examinations (pre and post treatment) could be analyzed.

In order to delineate MTVs on both pre and post treatment PET images, we considered in this study a relative threshold segmentation algorithm. Despite not being the most robust segmentation method, our choice should be considered within the context of analyzing only paired acquisitions performed on the same scanner using the same acquisition and reconstruction protocols. In addition, this approach allowed us to define multiple MTVs according to the variable intra-tumor uptake intensity. In agreement with the literature, using the most routinely used fixed threshold intensity (40% of the SUV_max_), the baseline MTV and TLG were higher in patients with a local recurrence (*p* = 0.0005) [20–22]. This finding confirms that initial MTV and TLG could help identifying patients whose local control would be insufficient and would therefore potentially benefit from a treatment intensification. In our cohort, tumor uptake was high (mean SUV_max_ of 14.3 ± 4.5) and all lesions were larger than 3 cm^3^, so theoretical overestimation of lesion size induced by the fixed threshold methodology was not necessarily an issue in this patient group.

To go further towards an efficient integration of functional imaging into the treatment planning, the use of the baseline 18FDG-PET images to identify intra-tumor areas with high recurrence risk was investigated in some cancer models such as esophageal or non small cell lung cancer and has shown promising results [13, 23, 24]. Due *et al.* have suggested that the highest metabolic area of the MTV in the baseline images is more likely to be the preferential site of local recurrence [25]. Calais *et al.* proposed a 70% SUV_max_ threshold to delineate the area having a high probability of recurrence to consider for potential dose escalation in NSCLC [21]. Similarly, the same group suggested a 60% SUV_max_ threshold to delineate the same area of interest in the case of esophageal cancer [10]. In a similar study, Lu *et al.* reported overlapping residual lesions in only 50% of the patients with an overlap fraction being low and very low considering identified higher FDG uptake sub-volumes [26]. In head and neck cancer, Soto *et al.* have reported results on a very small cohort of only 9 patients with a recurrence after concomitant radiochemotherapy. In 8 out of 9 patients local recurrence occurred within the initial hypermetabolic volume identified on the baseline FDG PET images [9]. Finally, a recent study performed in 19 patients reported that considering a 50% SUV threshold on initial PET images led only to a moderate agreement with the relapsing volume [14]. In our study, we have also found substantial overlap between initial and recurrent MTVs. However, the concordance between the initial metabolic sub-volumes (I_x_) and recurrence MTVs (R40 or R90) were also lower than those described on previous works performed on different cancer models [13, 24]. This difference may be the result of several factors. Firstly, all of MTVs we had in this HNSCC population were smaller by over a factor of two than those previously analyzed in either esophageal or lung cancer. For example, Calais *et al.* reported initial I_40_ MTVs of 53 cm^3^ in lung cancer while in our HNSCC study mean I_4O_ was only 24.6 cm^3^ [24]. The same discrepancy occurred when considering recurrence MTVs.

Secondly, Calais *et al.* have found the recurrences to mainly occur into the initial area for esophageal cancer patients using a similar approach to the one used in our study. The mean reported radiation dose of 50 Gy (against 69 Gy in our study) might suggest that the lower dose may have led to a more frequent regrowth into the initial tumor site. Indeed, actual recommendations are to use a minimum dose of 65 Gy on the gross tumor volume (GTV) and 50 Gy on the biological tumor volume, corresponding to the MTV in the case of using 18FDG-PET images, including microscopic tumor involvement for optimal local control [17]. Using a similar mean radiation dose to the one we have used in this study, Aerts *et al.* have reported a lower overlap between initial and recurrence volume in non-small cell lung cancer (OF > 0.6) [22]. Finally, in a heterogeneous HNSCC patient population, Chaput *et al.* also reported a lower overlap using a similar mean radiation dose of 69 Gy [14].

The remaining differences might be due to uncertainties associated with the coregistration of the sequential PET/CT images. In addition to morphological post treatment alterations, the accuracy of the rigid registration may be affected by patient repositioning between the two PET/CT acquisitions. Despite the potential limitations of a rigid registration, the use of a non rigid registration will reduce the relevance of intra-tumor volume distribution comparisons as a result of the induced tumor deformation. Amongst the previous studies available in HNSCC, the majority of them focused on a qualitative description of the recurrence sites, thus making the precision of the registration step less critical. Our present results highlight the important role of a standardized patient positioning when considering a quantitative comparison objective.

The use of an immobilization mask may improve the accuracy of the registration process. Due *et al.* have reported a high correlation between the gross tumor volume (GTV) and the recurrence site of advanced HNSCC treated by radiotherapy (IMRT) [25]. In this study patients were positioned and immobilized using radiotherapy masks that have obviously facilitated the registration process. Another group using a CT registration for HNSCC treated with IMRT has reported that the majority of the local relapses occurred within high dose regions [27]. However, in this study a deformable registration was also used and there is no information on the size of the overlap between recurrent MTVs and radiation planned volumes.

To assess and quantify the overlap between various metabolic sub-volumes derived from the baseline and follow-up PET images, we have used five different common indices. The Dice and Jaccard indices were the lowest in value. This can be explained by the important differences existing between the different sub-volumes considered. Indeed, these indices were directly dependent on the size of the compared volumes. The OF was higher than other indices when comparing the sub-volumes I_x_ and the recurrence volume R40, but it remained low (between 0.2 and 0.4 for I_30_ to I_70_). A small overlap was identified using V_cI_ , which was stable from I_30_ to I_80_. This result corresponds to a pattern of recurrence which was preferentially located at the edge rather than inside the baseline intra-tumor activity distribution.

None of the overlap indices have demonstrated an overlap between the initial sub-volumes I_x_ and the highest recurrence intensity area (R_90_). The indices used to quantify the concordance of the volumes depend strongly on the absolute compared volumes. In our cohort, all patients had much larger initial MTVs compared to very small R_90_ volumes (0.18 cm^3^), which can explain the fact that the different indices failed to quantitatively assess the overlap between R_90_ and the initial sub-volumes.

Our selection criteria limited the number of analyzed patients but led to a homogeneous cohort. Although the number of patients considered in this study is substantially larger than those used in previous studies with similar objectives, confirmation of our findings requires prospective trials which will include a standardized patient re/positioning protocol.

Our study has shown a low concordance between initial and recurrent metabolic sub-volumes determined by a fixed threshold method on a cohort of advanced HNSCC treated by concomitant radio-chemotherapy. This overlap, although small, reflected an insufficient local control for some patients occurring mostly at the edge of the baseline metabolically active tumor volume. The previously reported encouraging results in lung or esophageal cancer may not be directly translated in HNSCC, as already suggested by Chaput *et al.* in a small and heterogeneous group [14]. Although our results obtained in a larger and more homogeneous cohort need to be confirmed on an even larger patient cohort using a standardized patient repositioning methodology, they provide additional evidence that dose adaptation (boosting, escalation) using FDG PET in head and neck cancer is more complex than initially thought.

## MATERIALS AND METHODS

### Patients

94 patients with locally advanced HNSCC in the oral cavity or the oropharynx, referred to our institution between 2011 and 2016, were retrospectively included. All patients received radiotherapy as part of their treatment, and mostly (94.6%) concomitant radio-chemotherapy with a mean delivery dose of 69.1 ± 1.3 Gy on the gross tumor volume (2Gy per fraction). All patients underwent an initial 18F-FDG PET/CT scan before treatment at initial staging and after treatment during systematic follow up (4 months) or at the time of a clinically suspected recurrence. This study was validated by the ethics review board of our institution. All patient characteristics are summarized in the Table 3.

**Table 3:** Patient characteristics (*n* = 94) and detailed characteristics for patients with complete remission (CR) (*n* = 35), for patients with local recurrence (LR) or residual disease (RD) (*n* = 38) and for patients with lymph node or distance recurrence (RD) (*n* = 21)

	Total (*n* = 94)	CR (*n* = 35)	LR/RD (*n* = 38)	RD (*n* = 21)
Gender				
*Male*	80	30	34	16
*Female*	14	5	4	5
Age	59.2 ± 8.7	60.4 ± 10.4	58.8 ± 7.8	57.8 ± 7.4
Tumor site				
*oropharynx*	88	32	37	19
*Larynx*	2	1	0	1
*Oral cavity*	4	2	1	1
TNM				
*T1*	1	1	0	0
*T2*	16	8	5	3
*T3*	43	16	17	10
*T4*	34	10	16	8
*N0*	12	5	5	2
*N1*	9	1	5	3
*N2*	60	27	23	10
*N3*	13	2	5	6
Stage				
*II*	1	1	0	0
*III*	9	4	7	2
*IV*	84	30	31	19
Treatments				
*Radiotherapy mean dose*	69.1 ± 1.3	69.2 ± 2	68.3 ± 4	68.7 ± 1.8
*Radiotherapy mean time (days)*	36.0 ± 3.0	37.0 ± 3.0	35.2 ± 3.0	35.6 ± 2.0
*Radiochemotherapy*	81	29	31	21
*Radiotherapy alone*	5	2	3	0
*Induction chemotherapy followed by radiochemotherapy*	8	4	4	0

### 18F-FDG PET acquisition

18F-FDG PET images were acquired on a Biograph mCT 40 TOF (Siemens^®^, Erlangen, Germany) PET/CT system. Patients were required to fast for at least 6 hours prior to the PET/CT scans. Acquisitions were performed 60 minutes after the injection of 2.5 MBq/kg of 18F-FDG. The whole body acquisitions were performed with patient’s arms down while they were breathing freely. Non enhanced low-dose CT scans were acquired first for attenuation correction (120 kV, Care Dose^®^ current modulation system). PET acquisitions were performed from thigh to head (3.5 minutes per bed position). Images were reconstructed using CT-based attenuation correction and the OSEM-TrueX-TOF algorithm (3 iterations, 5 mm 3D Gaussian post-filtering). The reconstructed voxel size was 4 × 4 × 4 mm^3^.

### 18F-FDG PET image analysis

For each patient, the two PET/CT datasets were registered using an automatic rigid registration algorithm based on mutual information (Dosisoft^®^, France). More specifically, PET images were used prior to the registration to identify the area of interest (high uptake regions) and hence select the anatomical region to use on the registration of the two CT images. The deformations (translations + rotations) derived from the CT registration process were finally applied to the PET images. Post therapeutic PET/CT scans were registered on the corresponding baseline PET/CT scans which were systematically used as the reference image. On the baseline PET images, metabolic tumor sub-volumes (MTV), named I_x,_ were delineated using different fixed thresholds at a percentage of SUV_max_ and were *x* is the considered percentage. All thresholds from 30% to 90% with a step of 10% were considered. Tumor lesion glycolysis was also calculated on the baseline PET images by multiplying the MTV at 40% of SUV_max_ with the corresponding SUV_mean_ determined by considering this MTV.

On the post-therapeutic PET scans recurrence or residual disease were delineated using a threshold at 40% and 90% of the SUV_max_. These metabolic sub-volumes (MTV) called R_40_ and R_90_ aimed to respectively characterize the whole metabolic tumor volumes and the areas with the highest uptake as previously described [10, 21].

Five indices were used to evaluate the overlap between initial (I_x_) and recurrence (R_40/90)_) MTV; namely the Dice coefficient (D = 2.(l_x_∩R_40/90_)/(l_x_+R_40/90_)); Jaccard index (J = (l_x_∩R_40/90_)/(l_x_∪R_40/90_); overlap fraction (OF = (l_x_∩R_40/90_)/min(l_x_,R_40/90_)); common volume divided by I_x_ ((V_cI_ = l_x_∩R_40/90_)/l_x_); and common volume divided by R_40/90_ ((V_cR_ = l_x_∩R_40/90_)/R_40/90_).

The measurement of V_cI_ considering R_40_ determines the smaller initial sub-volume (I_x_) that contains the recurrence volume, while the measurement considering R_90_ corresponds to the smaller initial sub-volume (I_x_) that contains the highest activity regions in the tumor recurrence. Dice, Jaccard and OF indices are commonly used to compare volumes delineated by different methods or operators and are known to be robust to inaccuracies in the overlap of the compared volumes. OF leads to generally higher values due to the use of the smallest volume in the denominator, while Dice coefficients are known to be more sensitive to size differences between the two compared volumes. Theirs values may vary between 0 and 1, with 0 and 1 corresponding to no overlap and perfect match between the two compared volumes respectively.

### Statistical analysis

Quantitative data were characterized by their mean ± standard deviation, and by their range when the distribution was not normal (tested using an Agostino Pearson test). A Mann Whitney *U* test was used to compare I_40_ as derived from the initial scan in patients with complete response versus in patients with recurrent or residual disease. The same statistical test was used to compare the statistical significance between the baseline MTV and TLG of the two patient groups in terms of treatment response (relapse/residual disease vs complete response). *P*-values under 0.05 were considered as statistically significant.

The evaluation scale of the overlap between baseline and post-treatment scans was based on the methodology of Cohen *k*-test inter-observer agreement statistics: very low concordance for values between 0 and 0.2; low concordance for values between 0.21 and 0.4; moderate concordance for values between 0.41 and 0.60; good concordance for values between 0.61 and 0.80 and very good concordance for values between 0.81 and 1 [28].

## Abbreviations

18F-FDG: 18F-fluorodeoxyglucose
CT: Computed Tomography
GTV: Gross Tumor Volume
HNSCC: Head and Neck Squamous Cell Carcinoma
IMRT: Intensity-Modulated RadioTherapy
LR: Local Recurrence
MRI: Magnetic Resonance Imaging
MTV: Metabolic Tumor Volume
OF: Overlap Fraction
OSEM: Ordered Subset Expectation Maximization
PET: Positron Emission Tomography
SUV: Standardized Uptake Value
TNM: Tumor, lymph Nodes, Metastasis
TOF: Time Of Flight
